# Lipid Biosynthesis as an Antifungal Target

**DOI:** 10.3390/jof4020050

**Published:** 2018-04-20

**Authors:** Jiao Pan, Cuiting Hu, Jae-Hyuk Yu

**Affiliations:** 1 Department of Microbiology, College of Life Sciences, Nankai University, Tianjin 300071, China; 2120160931@mail.nankai.edu.cn; 2 Key Laboratory of Molecular Microbiology and Technology, Ministry of Education, Tianjin 300071, China; 3 Department of Bacteriology, University of Wisconsin-Madison, Madison, WI 53706, USA

**Keywords:** lipid metabolism, pathogenic fungi, antifungal drugs, target protein, lipidomics

## Abstract

Lipids, commonly including phospholipids, sphingolipids, fatty acids, sterols, and triacylglycerols (TAGs), are important biomolecules for the viability of all cells. Phospholipids, sphingolipids, and sterols are important constituents of biological membranes. Many lipids play important roles in the regulation of cell metabolism by acting as signaling molecules. Neutral lipids, including TAGs and sterol esters (STEs), are important storage lipids in cells. In view of the importance of lipid molecules, this review briefly summarizes the metabolic pathways for sterols, phospholipids, sphingolipids, fatty acids, and neutral lipids in fungi and illustrates the differences between fungal and human (or other mammalian) cells, especially in relation to lipid biosynthetic pathways. These differences might provide valuable clues for us to find target proteins for novel antifungal drugs. In addition, the development of lipidomics technology in recent years has supplied us with a shortcut for finding new antifungal drug targets; this ability is important for guiding our research on pathogenic fungi.

## 1. Introduction

The increasing number of immunocompromised patients has led to the emergence of many forms of fungal infections in recent years. The causative fungi are most commonly dermatophytic (*Epidermophyton*, *Microsporum*, or *Trichophyton* species) or saprophytic species (*Aspergillus*, *Blastomyces*, *Candida*, *Coccidioides*, *Paracoccidioides*, *Cryptococcus*, *Histoplasma*, or *Sporothrix* species). The saprophytes are also capable of causing systemic infections, which pose a great threat to human health under certain conditions [1]. Furthermore, a restricted arsenal of antifungals is available, and the development of resistance to antifungal drugs is increasing, which makes it imperative to discover new antifungal targets.

The existence of the cell wall in fungi is the most significant structural difference between fungi and mammalian cells. The main components of the fungal cell wall are β-1,3-glucan, chitin, and various glycoproteins, which are essential for the integrity of the cell wall and the maintenance of intracellular osmotic pressure. Therefore, disrupting the biosynthesis of the fungal cell wall has been an important strategy for the development of antifungal drugs. For example, the echinocandins, clinically important fungal inhibitors, are able to inhibit the synthesis of fungal β-1,3-glucan and thus abolish the integrity of the cell wall. Although the cell wall is important for fungi, from a lipid perspective, the plasma membrane is important as it contains the enzymes and proteins responsible for the cell wall biosynthesis [2,3]. In this review, we focus on the fungal metabolic pathways for lipids, including phospholipids, sphingolipids, fatty acids, and neutral lipids, and how the biosynthetic portions of these pathways differ from those of mammalian cells. Specific enzymes or proteins necessary for fungal lipid metabolism might have great potential to be antifungal targets. As important constituents of cellular membranes, lipids also have been proven to be targets of antifungals, which will be discussed here. Furthermore, some important lipid signaling molecules can regulate cell proliferation and/or cell death, which is tightly linked to the virulence and pathogenicity of the fungus. Thus, interference with these regulatory mechanisms may be an effective means of coping with fungal infections.

## 2. Classification and Function of Lipids

### 2.1. Classification of Lipids

**Figure 1:**
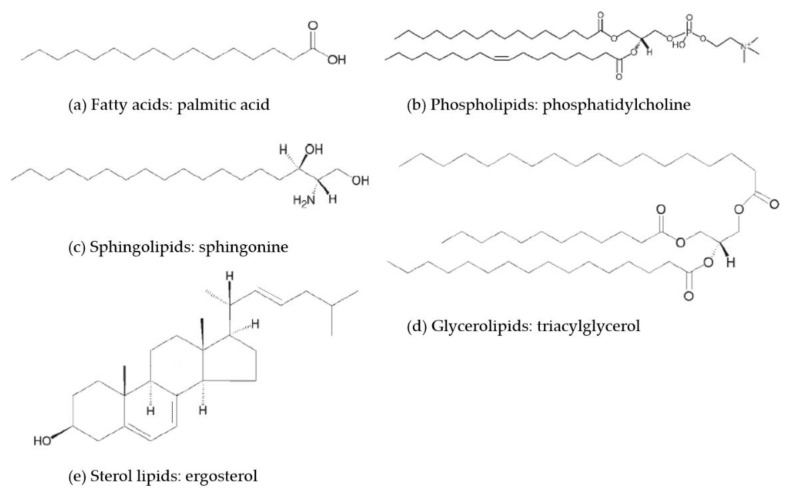
Representative structure of (**a**) fatty acids, (**b**) phospholipids, (**c**) sphingolipids, (**d**) glycerolipids, and (**e**) sterol lipids. This figure is adapted and modified from Ref. [4].

There are many kinds of naturally occurring lipid molecules with great differences in their chemical structures and physical properties. In 2005, to advance the development of lipidomics, the International Lipid Classification and Nomenclature Committee (ILCNC) classified lipids into eight categories: fatty acyls (FAs), glycerolipids (GLs), glycerophospholipids (GPs), sphingolipids (SLs), sterol lipids (STs), saccharolipids (SLs), prenol lipids (PRs), and polyketides (PKs) [4]. Among these categories, fatty acids, phospholipids, sphingolipids, glycerolipids, and sterols are common lipid molecules in cells (the basic structures of the main lipid groups are shown in Figure 1), while saccharolipids, prenol lipids, and polyketides are mainly found in bacteria, fungi, and plants [5].

Lipids are organic molecules that are insoluble in water but soluble in nonpolar organic solvents and have hydrophobic or amphiphilic properties; the physical properties of lipids determine their physiological function in cells. As important storage lipids in cells, triacylglycerols (TAGs) and sterol esters (STEs) are esterified forms of free fatty acids and sterols, respectively. These lipids are uncharged and less toxic to cells. Sphingolipids and phospholipids are amphiphilic lipids with fatty acyl chains that form hydrophobic cores and with the hydrophilic heads exposed to the environment; these types of lipids constitute the main backbone of cell membranes and, therefore, are important structural lipids in cells.

### 2.2. Function of Lipids

Of the total number of genes in cells, 5% are related to the metabolism of lipid molecules [6], not only reflecting the complex varieties of lipid molecules but also suggesting their important biological functions in cells. Therefore, it is conceivable that selective interference with the metabolism of lipid molecules can effectively inhibit the survival of pathogenic fungi in host cells.

First, lipid molecules are important for energy storage in cells. The β-oxidation of fatty acid chains provides a large amount of ATP, which allows other biological activities of cells to proceed properly. Fatty acids are the simplest lipid molecules in cells and are important building blocks of complex lipids such as phospholipids and sphingolipids. Surplus intracellular free fatty acids are stored in lipid droplets (LDs) [7] in the form of TAGs and STEs. LDs are not only an important energy reservoir for cells but are also an organelle closely related to lipid metabolism. A large number of hydrolases involved in the degradation of TAGs and STEs are located on the membrane and mobilize the decomposition of these two nonpolar lipids when needed [8].

Second, the glycerophospholipid bilayer constitutes the basic skeleton of the cell membrane, which isolates the cell from the external environment and ensures the independence of biochemical reactions as well as the formation of internal compartments in which different biological functions can be completed without interference. Glycerophospholipids can be further divided into phosphatidylcholine (PC), phosphatidylethanolamine (PE), phosphatidylinositol (PI), phosphatidylserine (PS), and so on, based on their head groups. In eukaryotic cells, the content and types of phospholipids vary in the membranes of different organelles and the plasma membrane. The major phospholipids in total cell extracts from *Saccharomyces cerevisiae* are PC, PE, PI, and PS (Table 1) [9]. In addition to glycerophospholipids, sphingolipids and sterols are also important components of cell membranes. They aggregate dynamically to form lipid rafts, which regulate downstream signaling molecules and increase the efficiency of signal transduction [10]. Lipid rafts also play an important role in pathogenicity by containing many of the virulence factors of fungi. In *Cryptococcus neoformans*, Pma1 is a proton pump that maintains the intracellular pH of fungal cells in the physiological range. Pma1 is a raft-localized protein and its association with rafts is required for surface delivery and protein stability in the plasma membrane. Mutants of *C. neoformans* in which Pma1 was not functioning properly showed attenuated virulence, likely due to their inability to survive in the acidic environment of the phagolysosome [11]. Moreover, sterols are able to regulate the fluidity and permeability of cell membranes. The structural function of lipid molecules in cells is irreplaceable. Recent studies in HeLa cells have shown that the fraction of lipids such as PI and PC in membrane systems changes during the critical phases of the cell cycle. It was shown that 11 kinds of lipids, for example, phosphatidic acid (PA) (16:0/16:0), diacylglycerol (DAG) (16:1, 12:0, 18:1), and PS (18:0/20:4) accumulate in dividing cells. The composition and localization of lipids are remodulated during the cell cycle. Therefore, the lipid composition of the cell membrane structure itself is very important to the cell [12,13].

Finally, lipids such as fatty acids [14,15], sterols [16], phospholipids [17], and sphingolipids [18] are important signaling molecules in cells and participate in intracellular signal transduction. Inositol 1,4,5-triphosphate and di-acyl-glycerol (DAG), which originate from phosphatidylinositol 4,5-diphosphate, are very important signaling molecules. IP_3_-Ca^2+^-DAG-dependent protein kinase C (PKC) phosphorylates numerous downstream proteins; the downstream mitogen-activated protein kinase (MAPK) cascades have important regulatory functions for cell wall integrity and oxidative stress responses [19,20]. DAG is also a key intermediate lipid metabolite and is involved in the regulation of lipid metabolism [16]. In response to the catalytic activity of different kinds of phospholipases, PC can be cleaved into various second messenger molecules such as lysophosphatidylcholine (LPC), phosphatidic acid (PA), and arachidonic acid (AA). These signaling molecules are responsible for the regulation of many biological processes within the cell [18]. Furthermore, ceramide (Cer), sphingosine-1-phosphate (S1P), glycosphingolipids (GSLs), and other sphingolipid molecules are involved in vital activities such as cell proliferation, differentiation and apoptosis, protein synthesis, glucose metabolism, and stress response [21].

Polyketides, lipid molecules that are not present in all eukaryotic cells, might be unfamiliar because they are not commonly thought of as lipids but rather as secondary metabolites in microorganisms or plants. However, polyketides are of great significance for the virulence of pathogenic fungi. Dihydroxynaphthalene is a multi-polyketide compound in *Aspergillus fumigatus*; the gene encoding the responsible polyketide synthase (PKS) is essential for the synthesis of dihydroxynaphthalene, and the knockout of *PKS* resulted in a pigmentation deficiency in the asexual spores. These pigment-free conidia are more susceptible to engulfment by immune cells [22]. Therefore, melanin is an important virulence factor in *A. fumigatus* [23]. The targeting of melanin production is an effective antifungal means for weakening the virulence and pathogenicity of pathogenic fungi.

The energy storage, signaling, and structural roles described above are three common functions of lipid molecules in almost all organisms. The disruption of lipid metabolism leads to the collapse of the entire cellular system. However, when we explore the antifungal targets in pathogenic fungi, both the inhibitory effect on fungi and the damage caused to the host need to be considered. Therefore, we summarize specific metabolic pathways within fungi that differ from those in mammalian cells and thus have the potential to be antifungal drug targets. The study of lipid function in eukaryotic cells instructs us in the exploration of effective mechanisms for fighting pathogenic fungi.

## 3. Specific Lipid Metabolism in Fungi

### 3.1. Metabolism of Ergosterol in Fungi

**Figure 2:**
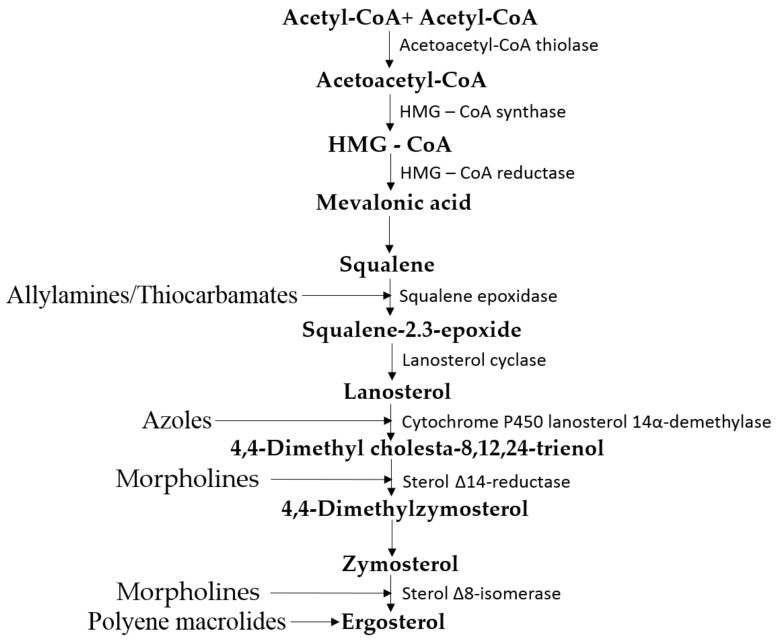
Antifungal interference with ergosterol biosynthesis. This figure is adapted and modified from Ref. [24]. HMG-CoA, β-Hydroxy-β-methylglutaryl-CoA.

The most important sterols in fungal and mammalian cells are ergosterol and cholesterol, respectively. Inhibiting the synthesis of ergosterol in fungi not only results in the structural disruption of the fungal cell membrane but also interferes with the functional organization of lipid rafts. The interaction of lipid rafts with proteins in a distinct biophysical environment may change the raft-localized proteins’ conformation and bioactivity—Pma1 in *C. neoforman*, for example. It can thereby cause great damage to fungal cells with minimal effects on human cells. The inhibition of ergosterol synthesis is the mode of action of many antifungal drugs, such as the azoles, the allylamines, and the morpholines. As shown in Figure 2, different drugs interfere with the specific process of ergosterol biosynthesis in *Saccharomycetes* [24]. The azoles have an inhibitory effect on cytochrome P450-dependent lanosterol 14α-demethylase, the allylamines and thiocarbamates inhibit squalene epoxidase, and amorolfin and other morpholine drugs have inhibitory effects on sterol Δ14-reductase and sterol Δ8,Δ7-isomerase. Distinct from these, the polyene macrolides, such as amphotericin B, are able to bind ergosterol in the fungal cell membrane, thereby forming hydrophilic channels that change the permeability of the cell membrane and resulting in the outflow of intracellular contents [25]. These drugs are clinically important inhibitors for the treatment of infection induced by pathogenic fungi. However, the development and use of these antifungal drugs are accompanied by several issues: (i) toxic side effects to the human body are easily produced; for example, amphotericin B inhibits fungi effectively with a broad-spectrum antifungal effect but is more toxic to the liver and kidneys in humans than are other drugs [26]; (ii) pathogens easily become tolerant to drugs, especially the azoles, which just cause temporary damage to pathogenic fungi but are not able to kill them directly, allowing the pathogens to rebound easily and develop antifungal resistance; and (iii) some of these drugs cannot be taken orally: the use of the echinocandins does not readily result in fungal resistance, and these drugs are less toxic to patients, but they must be administered intravenously [1]. The emergence of liposomal formulations of amphotericin B and combined antifungal therapy is destined to solve these limitations and expand the clinical applications of these drugs [27,28,29,30].

### 3.2. Metabolism of Phospholipids in Fungi

**Figure 3:**
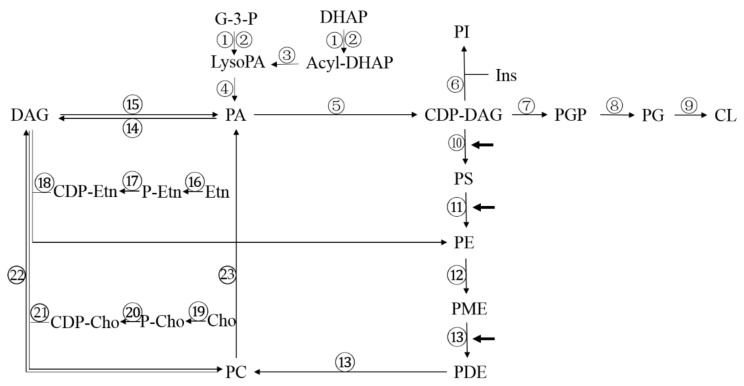
Metabolic network of phospholipids in *S. cerevisiae.* G-3-P, Glycerol-3-phosphate; DHAP, Dihydroxyacetone phosphate; PA, Phosphatidic acid; Lyso-PA, Lyso- Phosphatidic acid; Acyl-DHAP, Acyl-Dihydroxyacetone phosphate; CDP-DAG, Cytidine diphosphate-diacylglycerol; PI, Phosphatidyl inositol; Ins, Inositol; PGP, Phosphatidylglycerol phosphate; PG, Phosphatidylglycerol; CL, Cardiolipin; PS, Phosphatidylserine; PE, Phosphatidylethanolamine; PME, Phosphatidyl monomethylaminoethanol; PDE, Phosphatidyl dimethylaminoethanol; PC, Phosphatidylcholine; DAG, Diacylglycerol; Etn, Ethanolamine; P-Etn, Phospho-ethanolamine; CDP-Etn, Cytidine diphosphate-ethanolamine; Cho, Choline; P-Cho, Phospho-choline; CDP-Cho, Cytidine diphosphate-choline.

Phospholipids, which are the most abundant lipids in cells, comprise 40–60% of lipids in eukaryotic cells [6]. The metabolism of phospholipids in cells is very complicated, and there are substantial differences in the metabolic pathways in fungal and mammalian cells. It is necessary to clarify the metabolic network of phospholipids in fungal cells (Figure 3). The numbers in Figure 3 represent the catalytic enzymes in each reaction. Details of the enzymes are listed in Table 2.

The anabolism of phospholipid molecules begins with PA. The de novo synthesis of PA is via the two-step acylation of glycerol 3-phosphate (G-3-P) or dihydroxyacetone phosphate (DHAP) (① and ②). CDP-DAG (Cytidine diphosphate-diacylglycerol) synthase catalyzes the reaction between the substrates PA and cytidine triphosphate (CTP) that synthesizes CDP-DAG (⑤), which is the donor of the phosphatidyl group. Under the action of the corresponding enzymes, inositol (Ins), glycerol 3-phosphate, and serine act as head groups in the generation of PI (⑥), phosphatidylglycerol phosphate (PGP) (⑦), and PS (⑩), respectively. PGP is dephosphorylated by the action of phosphatase to form phosphatidylglycerol (PG), and PG is condensed with a molecule of CDP-DAG to form cardiolipin (CL) (⑨). In *S. cerevisiae*, PS is an important substrate for PC synthesis. PS is converted to PE by phosphatidylserine decarboxylase (⑪) and then synthesized through a three-step methylation reaction that is dependent on S-adenosylmethionine. The first step in the methylation reaction is catalyzed by phosphatidylethanolamine methyltransferase (⑫), encoded by *CHO2*, and the latter two steps are catalyzed by the *OPI3*-encoded phospholipid methyltransferase (⑬); this pathway is the endogenous pathway for the synthesis of PC driven by methylation reactions and is the main source of PC in *S. cerevisiae*. An alternative pathway for PC synthesis is the Kennedy pathway, in which exogenously obtained ethanolamine (Etn) or choline (Cho) are phosphorylated to generate phosphoethanolamine (P-Etn) (⑯) or phosphocholine (P-Cho) (⑲), respectively, under the action of the corresponding kinases. P-Etn or P-Cho binds to a single molecule of CTP to generate cytidine diphosphate ethanolamine (CDP-Etn) (⑰) or cytidine diphosphocholine (CDP-Cho) (⑳), respectively; these reactions are catalyzed by a cytidylyltransferase, and CDP-Etn and CDP-Cho finally react with DAG to form PE (⑱) or PC (㉑), respectively. In the reverse reaction, PC can be decomposed into DAG and PA under catalysis by phospholipase C (PLC) (㉒) and phospholipase D (PLD) (㉓), respectively [31].

PS synthase (⑩), PS decarboxylase (⑪), and phospholipid methylase (⑬), shown with bold arrows in Figure 3, have been reported in the literature as potential antifungal targets [32]. The *Candida albicans* PS synthase mutant, Δ*cho1*, lacking PS, has a decreased PE content and is avirulent in a mouse model of systemic candidiasis. The Δ*cho1* mutant exhibits defects in cell wall integrity, mitochondrial function, and filamentous growth. In addition, Cho1 does not have a homolog in mammalian cells; therefore, the inhibition of Cho1 activity is likely to become a new basis for antifungal therapy. Cho1 is conserved in fungi, and its inhibitors may have a broad-spectrum antifungal effect [33,34,35]. PS is a substrate for the de novo synthesis of PE. The *C. albicans* PS decarboxylase cannot be expressed at all when both *psd1* and *psd2* are knocked out. The resulting phenotype is similar to that resulting from *cho1* deletion, including reduced PE content and cell wall defects, but the decrease in virulence resulting from the former mutation is not as significant as that resulting from the latter [34]. In *Aspergillus nidulans*, the loss of *choC* (*opi3* in yeast) results in severely impaired vegetative growth, swelling of the hyphal tips, and the complete blockage of asexual and sexual development. This enzyme also has no homologous protein in the human body. Therefore, ChoC has a potential to be a drug target in the treatment of aspergillosis, although the alveolar surface may provide an alternative substrate for the synthesis of phospholipids by pathogenic fungi [35]. A study in the entomopathogenic fungus *Metarhizium robertsii* found that the deficiency of PE methylase (⑫) reduced the PC content of cells and impaired the production of conidia but had no significant effect on virulence. The reason that most enzymes related to the methylation reactions in the synthesis of PC can be potential antifungal targets is that the endogenous pathway is essential for the synthesis of PC in fungal cells, while PC is mainly synthesized via the Kennedy pathway in the cells of higher eukaryotes. In humans, only 30% of PC is synthesized via the methylation pathway in liver cells, a much higher percentage than in other organs [8,36,37].

### 3.3. Metabolism of Sphingolipids in Fungi

**Figure 4:**
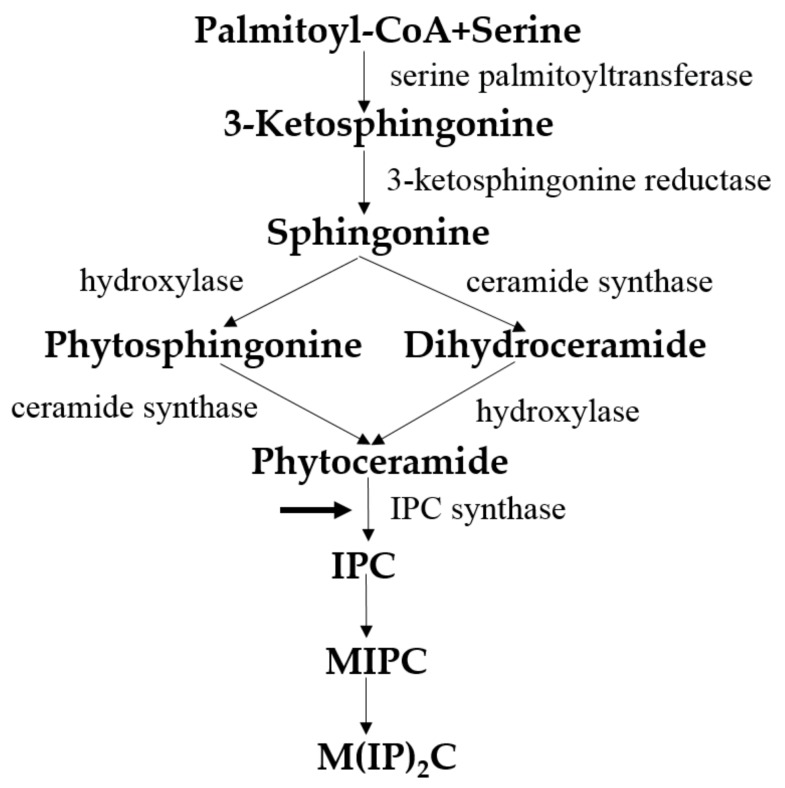
De novo synthesis of sphingolipids in *S. cerevisiae*. This figure is adapted and modified from Ref. [38]. IPC, inositolphosphatyl-ceramide; MIPC, mannose inositol-P-ceramide; M(IP)_2_C, mannose-(inositol-P)2-ceramide.

Sphingolipids, which are important components of cell membranes and act as signaling molecules in cells, are a complex family of compounds that share a common structural feature, a backbone of sphingoid bases that is synthesized de novo from serine and a long-chain fatty acyl-CoA. Condensation with fatty acids via amide bonds and/or hydroxylation at position C1 of the sphingoid base gives rise to the complexity and diversity of sphingolipid molecules. Figure 4 shows the de novo synthesis of sphingolipids in *S. cerevisiae* [34]. The synthesis of sphingolipids begins with a reaction between palmitoyl-CoA and serine; this reaction is catalyzed by serine palmitoyl transferase (SPT) and generates 3-ketodihydrosphingosine, which is reduced to dihydrosphingosine in an NADPH-dependent manner. Dihydrosphingosine is converted to phytosphingosine by the action of hydroxylase and ceramide synthase, and then phytoceramide binds a molecule of PI to synthesize inositolphosphatyl-ceramide (IPC) through a reaction catalyzed by IPC synthase. IPC can either be further mannosylated or condensed with PI to generate the more complex sphingolipids MIPC and M(IP)_2_C, respectively.

Most enzymes required for the de novo synthesis of sphingolipids have homologous proteins in mammalian cells; therefore, these enzymes cannot be used as targets for antifungal drugs because of their poor selectivity. Surprisingly, IPC synthase is a specific catalytic enzyme in fungi, which is not present in the human body; therefore, it has potential to become an antifungal target [38]. Furthermore, some antifungals have already been reported to effectively inhibit pathogenic fungi. Rustimicin 48 and galbonolide B have an inhibitory effect on the IPC synthase of *Cryptococcus neoformans* [39]. Khafrefungin 50 has exhibited different inhibitory effects on sphingolipid synthesis in *C. albicans*, *S. cerevisiae*, and *C. neoformans*. These inhibitors of IPC synthase show a strong antifungal effect and excellent selectivity, but they are not active against *A. fumigatus*; thus, the structural remodulation of these inhibitors may broaden their antifungal spectrum [40].

### 3.4. Metabolism of Fatty Acids in Fungi

Fatty acids, which act as building blocks for complex lipids such as phospholipids and sphingolipids, as well as energy reservoirs and signaling molecules, are the most fundamental lipid molecules in cells. Fatty acid molecules have a common basic structure with specific diversity determined by the chain length and the degree of unsaturation, and FAs are rapidly metabolized [4]. In yeast cells, acetyl-CoA, derived either from citric acid degradation or from acetate, is carboxylated into malonyl-CoA under the action of acetyl-CoA carboxylase; this reaction is the first step in the de novo synthesis of fatty acids and occurs in the cytoplasm or in mitochondria. The enzyme catalyzing the carboxylation reaction in the cytoplasm is encoded by *ACC1*, whereas the acetyl-CoA carboxylase in mitochondria is encoded by *HFA1*. FA synthesis is carried out in the cytosol by the hexameric fatty acid synthase (FAS) complex consisting of six Fas1p and six Fas2p subunits, each of which exhibit more than one enzymatic function. In contrast, the mitochondrial FA synthase complex consists of six proteins, each having only one enzymatic activity. Both the cytosolic and the mitochondrial FAS machineries add two carbons from malonyl-CoA to a saturated acyl chain. While the mitochondrial FAS system primarily produces lipoic acid, a chain length of up to 18 carbons can be produced by the cytosolic FA synthase. Further elongation to form very long chain FA (VLCFA) of up to 26 carbons in length by the stepwise addition of two carbons from malonyl-CoA is carried out in the endoplasmic reticulum (ER) by different elongases. Desaturation and hydroxylation of FA also occur in the ER [9].

The fatty acid synthase of the Basidiomycete yeast *C. neoformans* is composed of two subunits encoded by the *FAS1* and *FAS2* genes. The suppression of Fas1 and Fas2 expression blocks the de novo synthesis of fatty acids and severely inhibits vegetative growth of *C. neoformans*. However, supplementation with exogenous fatty acids was not able to rescue cell death induced by *FAS* deficiency. The treatment of *C. neoformans* with fluconazole was shown to have an increased inhibitory activity and to even become fungicidal when expression of either *FAS1* or *FAS2* was suppressed. In addition, Fas1 and Fas2 are different from the fatty acid synthase in the human body and thus may be potential antifungal target proteins [41].

### 3.5. Metabolism of Neutral Lipids in Fungi

Neutral lipids in fungi primarily include TAGs and STEs, which are responsible for the storage of free fatty acids, sterols, and DAG. DAG originating from the breakdown of PA serves as the primary backbone for the synthesis of TAG. The last step of de novo TAG formation in yeast can be accomplished through different routes. Lro1p, a homolog of the human lecithin-cholesterol acyltransferase (LCAT), catalyzes the transfer of an acyl group from the sn-2 position of PE or PC; this activity appears to be restricted to the ER. In an acyl-CoA-dependent reaction, the activated fatty acid can be linked to the glycerol backbone of DAG by Dga1p, which is localized to the ER and to LDs [42]. Are1p and Are2p, located in the ER, also have a minor role in the conversion of DAG to TAG, which also depends on acetyl-CoA. These two isomerases have 16% and 17% homology, respectively, with human acetyl-CoA:cholesterol acyltransferase (ACAT). Are1p and Are2p are two essential STE synthases in *S. cerevisiae*. Lipid analysis of Δ*are1* and Δ*are2* deletion strains revealed that Are2p and Are1p utilize sterol substrates in vivo with different efficiency; Are2p has a significant preference for ergosterol as a substrate, whereas Are1p esterifies sterol precursors (mainly lanosterol, as well as ergosterol). Nevertheless, terbinafine, an inhibitor of ergosterol biosynthesis, inhibits growth of Δ*are1*Δ*are2* cells more efficiently than growth of wild-type cells. In a growth competition experiment, Δ*are1*Δ*are2* cells grow more slowly than wild-type cells after several rounds of cultivation, suggesting that Are1p and Are2p or STEs, the product formed by these two enzymes, are more important in the natural environment than under laboratory conditions [43].

It is not certain whether any protein involved in the synthesis of neutral lipids has potential to be an antifungal target. However, the relationship between autophagy and TAG has already been reported in *Metarhizium robertsii*. It has been shown that nitrogen starvation is able to induce TAG accumulation in cells and activate autophagic activity but inhibits the internalization of LDs into vacuoles for degradation. Proteomic analysis of LDs identified an array of differentially accumulated proteins, including autophagy-related (ATG) proteins, heat shock proteins, and enzymes involved in TAG metabolism and phospholipid biosynthesis, in response to fungal growth under different nutrient conditions. This suggests that TAG metabolism and cellular nitrogen starvation are tightly related; however, the responsible mechanism is not yet clear. Further research in this field might provide new insights into the exploration of antifungal targets [44].

## 4. Searching for Potential Antifungal Drug Targets

The development of modern biotechnology, high-throughput sequencing, and chromatography and mass spectrometry for the separation and identification of biomolecules, as well as the cross-convergence of multiomics, have enabled new insights in the search for potential antifungal drug targets.

In 2016, Martin, K et al. combined three techniques for the discovery of anti-*A. fumigatus* targets: (i) modeling metabolic networks by elementary mode analysis and flux estimate approximations incorporating expression data from biochemical databank KEGG (Kyoto Encyclopedia of Genes and Genomes) and Metacyc [45,46,47]; (ii) targeting metabolic genes by transcriptome analysis to identify condition-specific highly expressed enzymes [48,49,50]; and (iii) analyzing enzyme structure and interconnectedness (“hubs”) and identifying pathogen-specific enzymes using orthology relationships. Sixty-four target candidates were finally obtained. The potential antifungal target proteins associated with lipid metabolism that have been reported in the literature are listed in Table 3. The first five genes/proteins are responsible for PC synthesis via the methylation pathway and the fatty acid synthesis pathway.

Pho88 (AFUA_5G01960) is an inorganic phosphate transporter that is involved in phospholipid synthesis in *A. nidulans*, *A. fumigatus*, and other *Aspergillus* species [51]. The homologous gene of AFUA_3G12320 in yeast is *Lpl1*, which encodes a phospholipase B; *Lpl1* is essential for the morphology of lipid droplets in yeast cells but is dispensable for cell survival because of the presence of other phospholipase B enzymes. However, from a clinical point of view, a phospholipase B blocker would be considered an interesting drug, because such a drug would probably block all phospholipase B activity in general, resulting in lethality [52]. In *S. cerevisiae*, Vtc4p is required during the fusion of inorganic phosphate-containing vesicles to the vacuolar membrane and the consequent accumulation of phosphate stored as polyphosphate (polyP) in the vacuole. In the maize pathogen *U. maydis*, Δ*vtc4* mutants were found to have reduced virulence in maize seedlings [53]; however, the loss of Vtc4 in *Candida* blocks the synthesis of polyP and does not lead to an enhanced sensitivity to oxidative stress [54]. Therefore, further studies are needed to determine whether this protein can be considered an antifungal drug target.

## 5. Conclusions and Outlook

In this review, we summarize fungal lipid metabolic pathways in comparison with those of humans, and discuss the specificities on which the antifungal mechanisms of the currently existing and yet-to-be-developed fungal inhibitors are based. The increased number of immunocompromised patients as a result of HIV infection or organ transplantations has attracted more and more attention to studies in this field, especially in relation to the virulence and pathogenicity of pathogenic fungi including *C. neoformans*, *C. albicans*, and *A. fumigatus*; such investigations create a new vision for the treatment of fungal infections. Multiomic data are becoming increasingly useful and definite with the progress of biotechnology and the aid of computer technologies including the Metatool program, YANA software package Borland JBuilder 2005, R statistics software, and so on; these advances provide more accurate and faster technical methods for the exploration of new antifungal targets and are expected to drive antifungal research.

## Figures and Tables

**Table 1:** Phospholipid composition in *Saccharomyces cerevisiae*.

Cell Fraction	Mol % of Total Phospholipids						
	PC	PE	PI	PS	CL	PA	Others
Homogenate	51.0	25.0	11.4	5.1	3.7	1.1	2.7
Plasma membrane	11.3	24.6	27.2	32.2	nd	3.3	1.4
Endoplasmic reticulum	38.9	18.6	22.4	6.4	0.3	6.1	10.0
Mitochondria	33.4	22.7	20.6	3.3	7.2	1.7	10.1
Peroxisomes	39.8	17.4	22.0	2.5	2.7	6.1	10.5

This table is adapted and modified from Ref. [9]. PC, phosphatidylcholine; PE, phosphatidylethanolamine; PI, phosphatidylinositol; PS, phoaphatidylserine; CL, cardiolipin; PA, phosphatidic acid; nd, not discovered.

**Table 2:** Enzymes catalyzing phospholipids metabolism in *S. cerevisiae*.

Number	Enzyme	Abbreviation
1	G-3-P acyltransferase	SCT1
2	G-3-P acyltransferase	GPT2
3	1-acyl-DHAP reductase	AYR1
4	Lyso-phospholipid acyltransferase	SLC1/ALE1
5	CDP-DAG synthase	CDS1
6	PI synthase	PIS1
7	PGP synthase	PGS1
8	PGP phosphatase	GEP4
9	CL synthase	CRD1
10	PS synthase	CHO1
11	PS decarboxylase 1/2	PSD1/PSD 2
12	PE methylase	CHO2
13	Phospholipids methylase	OPI3
14	PA phosphatase	PAH1
15	DAG kinase	DGK1
16	Etn kinase	EKI1
17	P-Etn cytidylyltransferase	ECT1
18	Etn phosphotransferase	EPT1
19	Choline kinase	CKI1
20	P-cho cytidylyltransferase	PCT1
21	Choline phosphotransferase	CPT1
22	Phospholipase C	PLC
23	Phospholipase D	SPO14

**Table 3:** Antifungal target proteins related to lipid metabolism.

Gene	Annotation	Fungi Tested *	Reference
AFUA_4G13680	Phosphatidylserine synthase	*C. albicans*	[33]
AFUA_2G15970	Phosphatidylethanolamine *N*-methyltransferase	*S. cersvisiae* et al.	[55]
AFUA_1G09050	Methylene-fatty-acyl-phospholipid synthase	*A. nidulans*	[35]
AFUA_3G04210	Fatty acid synthase α subunit FasA	*C. neoformans*	[41]
AFUA_3G04220	Fatty acid synthase β subunit FasB	*C. neoformans* *C. parapsilosis*	[41,56]
AFUA_5G01960	Phosphate transporter Pho88	*A. nidulans* *A. fumigatus*	[51]
AFUA_3G12320	Lipase/Serine esterase	*S. cerevisiae*	[57]
AFUA_2G09040	Vacuolar transporter chaperone (Vtc4)	*Ustilago maydis* *Candida*	[53,54]

* “Fungi tested” means that fungi listed here are already well described in literature and the researchers have published evidence supporting them as promising drug targets.
